# Suicide Gene Therapy By Amphiphilic Copolymer Nanocarrier for Spinal Cord Tumor

**DOI:** 10.3390/nano9040573

**Published:** 2019-04-08

**Authors:** So-Jung Gwak, Jeoung Soo Lee

**Affiliations:** 1 Department of Bioengineering, Drug Design, Development and Delivery (4D) Laboratory, Clemson University, Clemson, SC 29634-0905, USA; plus38317@gmail.com; 2 Department of Chemical Engineering, Wonkwang University, 460, Iksandae-ro, Iksan, Jeonbuk 54538, Korea

**Keywords:** suicide gene therapy, non-viral gene delivery, ganciclovir, spinal cord tumor

## Abstract

Spinal cord tumors (SCT) are uncommon neoplasms characterized by irregular growth of tissue inside the spinal cord that can result in non-mechanical back pain. Current treatments for SCT include surgery, radiation therapy, and chemotherapy, but these conventional therapies have many limitations. Suicide gene therapy using plasmid encoding herpes simplex virus-thymidine kinase (pHSV-TK) and ganciclovir (GCV) has been an alternative approach to overcome the limitations of current therapies. However, there is a need to develop a carrier that can deliver both pHSV-TK and GCV for improving therapeutic efficacy. Our group developed a cationic, amphiphilic copolymer, poly (lactide-co-glycolide) -graft-polyethylenimine (PgP), and demonstrated its efficacy as a drug and gene carrier in both cell culture studies and animal models. In this study, we evaluated PgP as a gene carrier and demonstrate that PgP can efficiently deliver reporter genes, pGFP in rat glioma (C6) cells in vitro, and pβ-gal in a rat T5 SCT model in vivo. We also show that PgP/pHSV-TK with GCV treatment showed significantly higher anticancer activity in C6 cells compared to PgP/pHSV-TK without GCV treatment. Finally, we demonstrate that PgP/pHSV-TK with GCV treatment increases the suicide effect and apoptosis of tumor cells and reduces tumor size in a rat T5 SCT model.

## 1. Introduction

Intramedullary spinal cord tumors (IMSCT) constitute 8 to 10% of primary spinal cord tumors [1] and are uncommon neoplasms characterized by irregular growth of tissue found inside the spinal cord. These tumors cause significant neurologic morbidity and mortality [2], such as pressure on sensitive tissues and reduced function, resulting in pain, sensory changes, and motor deficits. Current treatments for spinal cord tumor include surgical therapy, radiotherapy, and chemotherapy [3,4,5]. However, these conventional therapies have limitations such as tumor cell survival that leads to high recurrence rate, as well as infiltration of tumors into the spinal cord [6,7]. Radiation therapy is limited by dose-related toxicity to the normal spinal cord and surrounding tissues, and systemic toxicity [8]. Chemotherapy has also been used to treat SCT [9], but it has dose-limiting toxicity and side effects such as sensory neuropathies and gastrointestinal disturbances after systemic administration [10,11].

Gene therapy has been investigated as an alternative approach to overcome the limitations of current therapies for SCTs [12,13]. Among gene therapies, suicide gene therapy has received considerable attention in the field of cancer gene therapy. Suicide gene therapy is based on the gene-directed enzyme prodrug strategy, which requires suicide genes and prodrugs. In gene-directed enzyme prodrug therapy, a viral or non-viral vector is used to transfect tumor cells with a gene encoding a specific exogenous enzyme. A prodrug is then administered that can be only activated by the specific enzyme expressed in the tumor cells. The Herpes simplex virus thymidine kinase (HSV-TK) gene is the most frequently used suicide gene with the anticancer prodrug, ganciclovir (GCV) [13,14,15,16]. Won et al. reported that reducible poly (oligo-D-arginine) (rPOA) could deliver plasmid encoding herpes simplex virus-thymidine kinase (pHSV-TK)and demonstrated that locally injected rPOA/pHSV-TK with systemically administered GCV significantly reduced tumor volume and improved locomotor function compared to the GCV only group and naked pHSV-TK with GCV-treated group [12,13]. One of the drawbacks of this pHSV-TK and GCV combination therapy is the GCV concentration in tumor cells. GCV has a very low oral bioavailability (~5%) and short plasma half-life (~2–6 h), so GCV has to be injected daily (5 mg/kg) to maintain the therapeutic plasma concentration. To improve GCV concentration in plasma, several delivery systems have been studied, including liposomes [17,18] and silicone pellets [19]. Kajiwara et al. reported that they achieved longer circulation of liposome encapsulated GCV (PEG-GCV-lipo) in blood and the area under the curve (AUC) of liposome encapsulated GCV (PEG-GCV-lipo) was 36-fold and 32-fold higher compared to GCV solution after intravenous and intraperitoneal injection in mice, respectively [18]. They reported that PEG-GCV-lipo was three times more effective than GCV solution in inhibiting tumor growth in a mouse KB xenografts model. Therefore, there is a need to develop a carrier that can efficiently deliver both pHSV-TK and GCV to improve the efficacy of this suicide gene therapy.

Our group developed a cationic, amphiphilic copolymer, poly (lactide-co-glycolide)-graft-polyethylenimine (PgP), as a drug and nucleic acid delivery carrier [20,21,22]. We reported its ability to efficiently deliver plasmid DNA (pDNA) and small interfering RNA (siRNA) in various cell lines and primary neurons in vitro, as well as in the normal rat spinal cord [20]. We showed that PgP loaded with a fluorescent, hydrophobic dye (DiR; 1,1′-Dioctadecyl-3,3,3′,3′-Tetramethylindotricarbocyanine Iodide) is retained within the injured spinal cord for up to five days after intraspinal injection. Injection of PgP complexed with siRNA targeting Ras homolog gene family, member A (RhoA) in the injured rat spinal cord resulted in RhoA knockdown, reduced astrogliosis and necrotic cavity formation, and increased axonal sparing/regeneration [21]. Finally, we reported that a hydrophobic drug, rolipram, can be encapsulated in the hydrophobic core of PgP, increasing its aqueous solubility seven times compared to that in water alone [22].

Our long-term goal is to develop PgP as a platform technology for delivery of therapeutic drugs and nucleic acids to spinal cord tumors. In this study, we investigated PgP as a suicide gene carrier. We show that PgP can efficiently deliver pDNA encoding green fluorescence protein (pGFP) in rat glioblastoma (C6) cells in vitro and deliver pDNA encoding β-galactosidase protein (pβ-gal) in a rat spinal cord tumor model in vivo. We demonstrate that PgP/pHSV-TK with GCV achieves a suicide effect in C6 cells in vitro. Finally, we show the suicide effect of PgP/pHSV-TK with GCV treatment in a rat T5 spinal cord tumor model.

## 2. Material and Methods

### 2.1. Plasmid Amplification and Purification

Plasmids encoding the Monster Green Fluorescent Protein (phMGFP Vector, pGFP, Promega, Madison, WI, USA), beta-galactosidase (pSV40-βGal, βGal, Promega), and herpes simplex virus-thymidine kinase (pHSV-TK, Invivogen, San Diego, CA, USA) were transformed into *Escherichia coli* DH5α (Life Technologies, Grand Island, NY, USA) and amplified in Lysogeny broth (LB) medium with ampicillin at 37 °C overnight with shaking at 250 rpm. The amplified plasmids were purified by the Endorsee Maxi plasmid purification kit (Qiagen, Valencia, CA, USA). The quantity of plasmid was determined by the absorbance at 260 nm and the quality of plasmid was determined by the ratio of 260/280 nm using BioTek Take 3 microplate reader (BioTek, Synergy HT, Winooski, VT, USA).

### 2.2. Particle Size and Surface Charge of PgP/pDNA Polyplex

PgP was synthesized using PLGA (lactide:glycolide 75:25, 25 kDa, 120 µmole, Durect Corporation, Pelham, AL, USA) and branched polyethylenimine (bPEI, MW: 25 kDa, 100 µmol, Sigma, Milwaukee, WI, USA) as previously published by our laboratory [22]. PgP/pGFP polyplexes were prepared by mixing PgP and pGFP at various N/P (number of nitrogen atoms in PgP/number of phosphorus atoms in DNA) ratio and incubated for 30 min at 37 °C. Polyplex size distribution was measured using dynamic laser light scattering and zeta potential was measured electrophoretically by Zeta PALS (Brookhaven Instruments Corp., Holtsville, NY, USA). The mean diameter and zeta potential of polyplexes were measured in triplicate.

### 2.3. Transfection Efficiency and Cytotoxicity of PgP/pDNA Complex in 10% Serum Condition

To evaluate PgP as a non-viral gene carrier, we first measured the transfection efficiency and cytotoxicity of polyplexes formed with PgP and reporter gene, *pGFP*, in rat glioma (C6) cells in vitro. C6 cells were maintained in DMEM/F12 supplemented with 10% FBS and 1% penicillin/streptomycin at 37 °C in 5% CO_2_. For transfection, the cells were seeded at a density 1 × 10^5^ cells/well in a 12-well plate and cultured overnight. The polyplexes of PgP/pGFP (2 µg pGFP) were prepared at various N/P ratios ranging from 15/1 to 60/1 and incubated for 30 min at 37 °C. Polyplexes (2 µg pDNA/well) were added to the cells in the media containing 10% FBS and incubated at 37 °C. At 24 h post-transfection, the media containing polyplexes were removed and the cells were washed and replenished with fresh media and then cultured for an additional 24 h. GFP-expressing cells were counted by easyCyte flow cytometer (Millipore, Darmstadt, Germany) and transfection efficiency is expressed as % transfected cells according to the following equation:

% transfection efficiency = (number of GFP-positive cells/number of total cells) × 100

Transfected cells were imaged using an inverted fluorescent microscope (Zeiss Axiovert 200, Göttingen, Deutschland). The bPEI/pGFP (N/P ratio 5/1) complex was used as a positive control.

Cytotoxicity was analyzed in parallel experiments using the MTT assay. At 48 h post-transfection, the medium was removed and cells were washed with PBS, and incubated with 1 mL of fresh medium containing 3-(4,5-dimethylthiazol-2-yl)-2,5-diphenyltetrazolium bromide (MTT, 2 mg/mL in PBS, Sigma, Milwaukee, WI, USA). After incubation for 4 h at 37 °C, the MTT solution was removed and 1.5 mL of dimethylsulfoxide (DMSO) was added to dissolve the formazan crystals formed by the live cells. Absorbance (A) was measured at 570 nm. The cell viability (%) was calculated compared to non-transfected control according to the following equation:

Cell viability (%) = (A_570(sample)_/A_570(control)_) × 100%

### 2.4. Characterization of PgP/pDNA Polyplexes

#### 2.4.1. Stability of PgP/pDNA Polyplex

To evaluate the stable complex formation of the PgP/pDNA polyplex, PgP/pGFP complexes were prepared at various N/P ratios in deionized water and incubated for 30 min at 37 °C. The complexes were loaded on a 1% (*w*/*v*) agarose gel and electrophoresed for 60 min at 80 V. The gel was stained with ethidium bromide for 30 min and then imaged on an ultraviolet (UV) illuminator (GELDOC-IT2 imager, UVP, Waltham, MA, USA) to visualize the retention of complexes and migration of naked pDNA.

#### 2.4.2. Heparin Competition Assay

The stability of the PgP/pGFP polyplexes was evaluated using a heparin competition assay. Briefly, PgP/pGFP at an N/P ratio of 60/1 was prepared as described above. Ten microliter aliquots of complex solutions were placed into tubes. Then heparin, a negatively charged polysaccharide, was added at 0–40 heparin/pDNA, *w*/*w* ratio and the polyplex/heparin solutions were incubated at 37 °C for 30 min. The samples were immediately analyzed by 1% agarose gel electrophoresis as described above.

#### 2.4.3. Stability of PgP/pDNA in Serum

To measure the stability of the PgP/pGFP polyplexes in serum, PgP/pGFP polyplexes were prepared at a N/P ratio of 60/1 and then incubated in the media containing 10% FBS at 37 °C. Naked pDNA incubated in the media containing10% FBS was used for comparison. At 30 min, and 1, 3, 6, 24, and 72 h post-incubation, the samples were evaluated by 1% agarose gel electrophoresis as described above.

### 2.5. Long-Term Storage Stability of PgP/pDNA Polyplexes

To evaluate stability during long-term storage, PgP/pGFP polyplexes at an N/P ratio of 60/1 were selected based on the results from in vitro transfection and cytotoxicity. PgP/pGFP polyplexes at an N/P ratio of 60/1 (2 µg pGFP) were prepared and incubated at 4 °C for 6 months. The stability of stored PgP/pGFP polyplexes was evaluated at predetermined time points (6 h, 1 day, 3 days, 1 week, and 1, 3, 4, 5, and 6 months) using 1% agarose gel electrophoresis and transfection efficiency in C6 cells as described above. Freshly prepared PgP/pGFP polyplexes were used as a control. GFP-positive transfected cells were imaged using an inverted fluorescent microscope (Zeiss Axiovert 200, Göttingen, Germany).

### 2.6. Suicide Effects of PgP/pHSV-TK Polyplex and GCV Treatment In Vitro

To evaluate the suicide effect of PgP/pHSV-TK polyplex with GCV prodrug, C6 cells were seeded in 12-well plates at 1 × 10^5^ cells/well and cultured for 24 h. PgP/pHSV-TK polyplexes (2 µg, pHSV-TK) were prepared at an N/P ratio of 60/1, added to the cells cultured in DMEM/F12 containing 10% FBS, and incubated at 37 °C. At 24 h post-transfection, the media were relaced with fresh media containing 2 different GCV concentrations (50 and 100 µg/mL). At 1 and 4 days post-GCV treatment, the anti-cancer efficacy of PgP/pHSV-TK with GCV was analyzed via the MTT assay as described above. PgP/pHSV-TK at the N/P ratio of 60/1 only, GCV (100 µg/mL) only, and bPEI/pHSV-TK at an N/P ratio of 5/1 with GCV (100 µg/mL) were used for comparison. We used PgP/pGFP at an N/P ratio of 60/1 as a control to eliminate the cytotoxicity of the polyplex itself.

### 2.7. Generation of Spinal Cord Tumor Model

All surgical procedures and postoperative care were conducted according to National Institute of Health (NIH) guidelines for the care and use of laboratory animals (NIH publication No. 86–23, revised 1996) and under the supervision of the Clemson University Institutional Animal Care and Use Committee. Sprague Dawley rats (250–300 g, male) were anesthetized using isoflurane gas and laminectomy was performed at the T5 spinous process using an orthopedic bone cutter. C6 glioma cells (1.0 × 10^6^ cells in 3 μL PBS) were injected into the T5 position using a Hamilton syringe (26 G) (Hamilton, Bonaduz, Switzerland) [12,13,23]. As a control, PBS was injected into the T5 spinal cord. After C6 cell injection, the paraspinal muscles and the skin were closed with 3–0 silk suture. After surgery, animals were warmed with a heating blanket for recovery. To verify the tumor formation, animals were euthanized 12 days post-tumor cell injection by CO_2_ overdose, and spinal cord tissue from the region surrounding the injection site was explanted. Samples were embedded in Tissue-Tek^®^ O.C.T compound (Sakura Finetek Inc., Torrance, CA, USA) on liquid nitrogen, sectioned, and stained with hematoxylin and eosin (H&E).

### 2.8. Transfection Efficiency of PgP/pβ-Gal in a Rat Spinal Cord Tumor Model In Vivo

To evaluate PgP as a gene carrier in vivo, we used pβ-gal instead of pGFP for transfection to avoid potential interference from tissue autofluorescence. Animals were injected with C6 glioma cells (1.0 × 10^6^ cells in 3 μL PBS) at T5 as described above. At 5 days post-injection, the rats were randomly assigned to one of three experimental groups: (1) PgP/ pβ-gal polyplexes (*n* = 4 rats/group), (2) bPEI/pβ-gal complex, and (3) naked pβ-gal. PgP/ pβ-gal polyplexes (20 µL, 10 µg pβ-gal) at an N/P ratio of 60/1 were injected into the spinal cord tumor using a Hamilton syringe (26 G). bPEI/pβ-gal complex (20 µL, 10 µg pβ-gal) at an N/P of ratio 5/1 and naked pβ-gal (20 µL, 10 µg pβ-gal) were injected for comparison. After injection, the paraspinal muscles and the skin were closed with 3-0 silk suture. Seven days after treatment, rats were sacrificed by cardiac perfusion with 4% para-formaldehyde (PFA) in saline. The retrieved spinal cords were embedded in Tissue-Tek^®^ O.C.T. compound and sectioned into 10-μm thickness using a cryostat (Lecia CM 1950, Leica, Wetzlar, Germany) for histological analyses. The sections were fixed with 4% PFA, washed two times in PBS (pH 7.4) for 5 min, stained using X-gal staining solution (Invitrogen, NY, USA) overnight, washed in distilled water, and then counter stained with eosin.

### 2.9. Suicide Effect of PgP/pHSV-TK Polyplexes with GCV in a Rat Spinal Cord Tumor In Vivo

To evaluate the suicide effect of PgP /pHSV-TK polyplexes with GCV in vivo, rats were randomly assigned to one of three experimental groups (*n* = 4/group): (1) PgP/pHSV-TK polyplexes with GCV, (2) PgP/pHSV-TK polyplexes without GCV, and (3) saline injection (Untreated) at 5 days after C6 cell injection, as described above. PgP/pHSV-TK polyplexes (10 µg pHSV-TK, 20 µL) at an N/P ratio of 60/1 were prepared and injected in the tumor lesion by Hamilton syringe (26 G ) and GCV (40 mg/kg) was administered by intraperitoneal (i.p.) injection every day for 10 days [13]. PgP/pHSV-TK polyplexes (10 µg pHSV-TK, 20 µL) at an N/P ratio of 60/1 without GCV treatment were used for comparison and saline-injected animals were used as the untreated control. At 10 days post-GCV treatment, the rats were sacrificed by cardiac perfusion with 4% PFA in saline. The spinal cords were explanted, embedded, and sectioned as described above. To measure the tumor size, 16 sections (4 sections/rat, 4 rats/group) were stained with H&E, imaged. Quantitative measurements of tumor area were analyzed by Image J. The percent tumor area was calculated according to the following equation:

% Tumor area = (Tumor area/Total area of spinal cord) × 100

To evaluate the suicide effect by PgP/pHSV-TK polyplex (N/P ratio of 60/1) with GCV, the terminal deoxynucleotidyl transferase dUTP nick end labeling (TUNEL) assay was performed to identify apoptotic cells. Briefly, the sections were stained by using the ApopTag Plus Fluorescein In situ Apoptosis Detection kit (Chemicon International, Temecula, CA, USA) and nuclei were counterstained by 4′,6-diamidino-2-phenylindole (DAPI) and digitally imaged using an inverted epifluorescence microscope (Zeiss Axiovert 200, Göttingen, Germany). The untreated spinal cord tumor group and the PgP/pHSV-TK polyplex (N/P ratio of 60/1) without GCV group were used for comparison. We also evaluated the suicide effect of the PgP/pHSV-TK polyplex (N/P ratio of 60/1) with GCV on expression of Bcl-2-associatied X protein (Bax), a pro-apoptotic protein. Briefly, sections were stained using antibody against Bax (1:200, sc-23959, Santa Cruz Biotechnology, Dallas, TX, USA), followed by Cy3-conjugated anti-rabbit IgG (1:200, ab97035, Abcam, Cambridge, MA, USA). The stained sections were counterstained with DAPI and imaged using an inverted epifluorescence microscope.

### 2.10. Statistical Analysis

Quantitative data are presented as the mean ± standard deviation. Statistical analysis was performed by one-way ANOVA with the least significant difference (LSD) method used for *post-hoc* comparisons between subgroups. A *p*-value less than 0.05 was considered significant.

## 3. Results

### 3.1. Characterization of PgP/pDNA Polyplexes

The particle size and zeta potential of PgP/pDNA polyplexes at various N/P ratios were evaluated. The mean size of the PgP/pDNA polyplexes at all N/P ratios was about 143.4 nm with narrow polydispersity. The negatively charged pDNA was completely neutralized by positively charged PgP and the surface charge of PgP/pDNA polyplexes at N/P ratio of 15/1 or above was positive (Table 1).

### 3.2. Transfection Efficiency and Cytotoxicity of PgP/pDNA Polyplexes in 10% Serum Condition In Vitro

**Figure 1:**
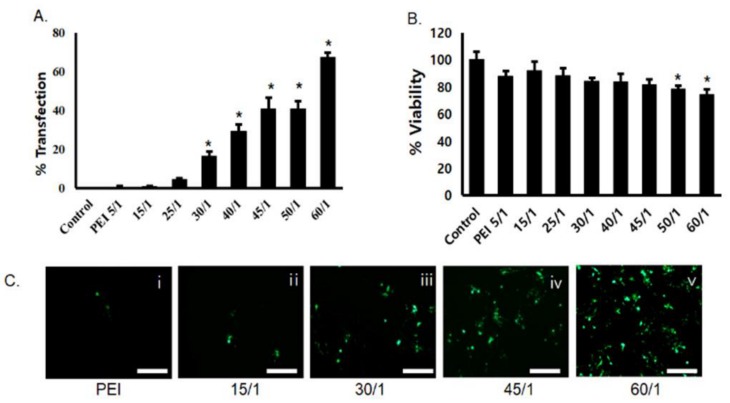
Transfection efficiency and cytotoxicity of PgP/pGFP polyplexes: (**A**) percent transfection efficiency and (**B**) percent cell viability after transfection of PgP/pGFP polyplexes in C6 cells in media containing 10% serum. Data represent the mean ± SD. (**C**) Representative images of GFP-positive cells after transfection with (i) bPEI/pGFP at N/P ratio of 5/1 and (ii–v) PgP/pGFP polyplexes at N/P ratios of 15/1, 30/1, 45/1 and 60/1. Scale bars indicate 100 μm.

Transfection efficiency of PgP/pGFP polyplexes was evaluated in C6 cells in media containing 10% serum. GFP expression increased with increasing polyplex N/P ratio and the highest transfection efficiency (69%) was observed at an N/P ratio of 60/1. In contrast, the transfection efficiency of bPEI/pDNA polyplexes at an N/P ratio of 5/1 was approximately 1% (Figure 1A). Cell viability was modestly lower for bPEI/pGFP and PgP/pGFP at all N/P ratios, but the difference was only statistically significant at N/P ratios of 50/1 and 60/1 (Figure 1B). Figure 1C shows representative images of GFP-positive cells after transfection with PgP/pGFP polyplexes at various N/P ratios. Based on the high transfection efficiency with low cytotoxicity, we performed all subsequent experiments using PgP/pDNA at an N/P ratio of 60/1.

### 3.3. Stability of PgP/pDNA Polyplex

**Figure 2:**
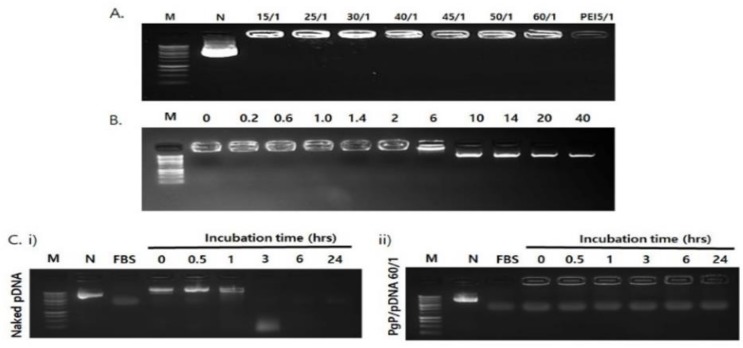
Analysis of polyplex stability. (**A**) Gel retardation assay of PgP/pDNA polyplexes at varying N/P ratios. Molecular weight marker (M, lane 1), naked pDNA (N, lane 2), PgP/pDNA polyplexes prepared at N/P ratios of 15/1, 25/1, 30/1, 40/1, 45/1. 50/1, and 60/1 (lanes 3–8) and bPEI/pDNA polyplex at N/P ratio of 5/1 (lane 9). (**B**) Heparin competition assay. PgP/pDNA polyplexes (2 μg pDNA) at N/P ratio of 60/1 were incubated with solutions containing heparin at varying concentration (0–40 heparin/pDNA, *w*/*w* ratio) at 37 °C for 30 min. M: Molecular marker. (**C**) Naked pDNA and PgP/pDNA polyplexes at N/P ratio 60/1 at various time points during incubation in 10% serum-containing media. (i) Naked pDNA and (ii) PgP/pDNA polyplex at N/P ratio 60/1, N is naked, untreated pDNA control.

The ability of PgP to condense pGFP was evaluated by gel retardation assay. Complete retardation of electrophoretic mobility was achieved at N/P ratios of 15/1 or above (Figure 2A). The stability of PgP/pGFP polyplexes was also evaluated by heparin competition assay. PgP/pDNA polyplexes at an N/P ratio of 60/1 that selected for highest transfection efficiency were prepared and incubated in solutions with varying heparin concentrations. PgP/pDNA polyplexes were stable in the presence of up to 6 *w/w* heparin/pDNA ratio and completely dissociated at ratios of 10 or higher (Figure 2B). The integrity of PgP/pDNA polyplexes at the N/P ratio of 60/1 in the presence of serum was evaluated after incubation in media containing 10% FBS. Naked pDNA incubated in the presence of serum was used for comparison. Naked pDNA was degraded by nucleases in the serum and undetectable after 3 h incubation (Figure 2Ci), whereas PgP/pDNA polyplexes were stable up to 24 h incubation (Figure 2Cii).

### 3.4. Long-Term Storage Stability of PgP/pGFP Polyplexes

**Figure 3:**
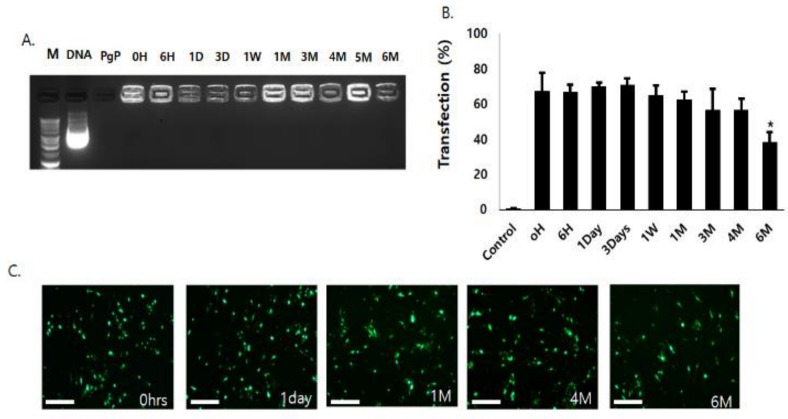
Long-term stability of PgP/pGFP polyplexes (2 μg pGFP, N/P ratio 60/1) at 4 °C. (**A**) Gel retardation assay by 1% agarose gel electrophoresis of PgP/pGFP polyplexes. M: Molecular weight marker (lane1), DNA: naked pDNA (lane 2), PgP only (lane 3), lanes 4–13: Polyplexes at pre-determined time points (0, 6 h, 1, 3, 7 days, 1, 3, 4, 5 and 6 months, respectively) during storage at 4 °C. (**B**) Transfection efficacy of polyplexes stored at 4 °C in C6 cells in media containing 10% serum at pre-determined time points. Data represent the mean ± SD. * *p* < 0.05 compared with freshly prepared polyplexes. (**C**) Representative images of GFP-positive cells at 2 days post-transfection with PgP/pGFP polyplexes stored 4 °C. Scale bars indicate 100 μm.

To evaluate long-term stability, PgP/pGFP polyplexes at N/P ratio 60/1 were stored at 4 °C for six months. Gel electrophoresis analysis showed that PgP/pGFP polyplexes were stable up to six months at 4 °C (Figure 3A). Transfection efficiency of PgP/pGFP polyplexes stored at 4 °C for up to four months was not significantly different compared to freshly prepared PgP/pGFP polyplexes, but significantly decreased after six months’ storage (Figure 3B). Figure 3C shows representative images of GFP-positive cells after transfection with PgP/pGFP polyplexes stored at 4 °C.

### 3.5. Suicide Effect of PgP/pHSV-TK Polyplex with GSV Treatment In Vitro

**Figure 4:**
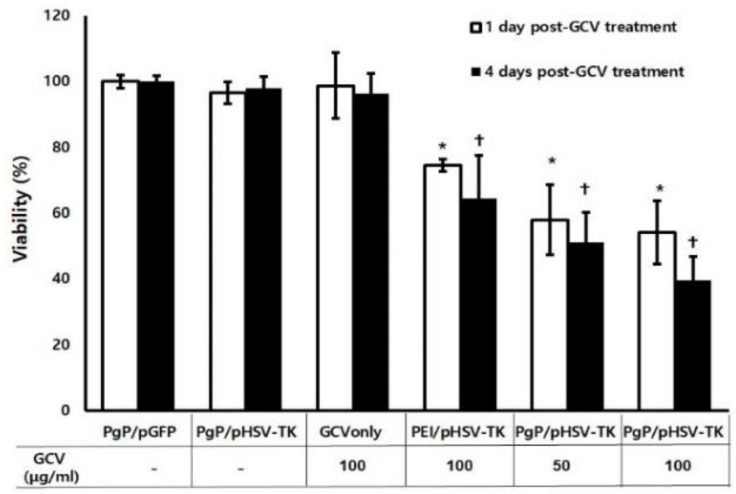
The suicide effect of PgP/pHSV-TK polyplexes and GCV. C6 cells were transfected with PgP/pHSV-TK (2 µg pHSV-TK) and then treated with GCV (50 and 100 µg/mL). At 1 and 4 days post-GCV treatment, anti-cancer efficacy was analyzed by MTT assay. PgP/pGFP (N/P of 60/1), PgP/pTK (N/P ratio of 60/1), GCV only (100 µg/mL), bPEI/pTK (N/P ratio of 5/1) with GCV (100 µg/mL) were used for comparison. Data represent the mean ± SD. *: *p* < 0.05 compared to PgP/pGFP at 1 day post-GCV treatment and **+**: *p* < 0.05 compared to PgP/pGFP at 4 days post-GCV treatment.

The suicide effect of PgP/pHSV-TK (N/P ratio of 60/1, 2 µg pHSV-TK) polyplexes with two different GCV doses (50 and 100 µg/mL) was evaluated at 1 and 4 days after GCV treatment in C6 cells. PgP/pGFP polyplexes (N/P ratio of 60/1, 2 µg pGFP) were used as a control instead of untreated cells to eliminate the cytotoxicity caused by the polyplex itself. PgP/pHSV-TK only or GCV (100 µg/mL) only, and PEI/pHSV-TK (N/P ratio of 5/1, 2 µg pHSV-TK) were used for comparison. PgP/pHSV-TK only or GCV only (100 µg/mL) did not show significantly different anti-cancer efficacy compared to PgP/pGFP polyplexes at both 1 and 4 days after treatment (Figure 4). The anti-cancer efficacy of PgP/pHSV-TK polyplexes with both GCV doses of 50 and 100 µg/mL was significantly higher than that of PgP/pGFP polyplexes at both 1 and 4 days post-treatment. We also observed that the anti-cancer efficacy of PgP/pHSV-TK polyplexes was significantly higher than that of bPEI/pGFP polyplexes (both with 100 µg/mL GCV) at 1 and 4 days post-treatment.

### 3.6. Transfection Efficiency of PgP/pβ-Gal in a Rat Spinal Cord Tumor Model In Vivo

**Figure 5:**
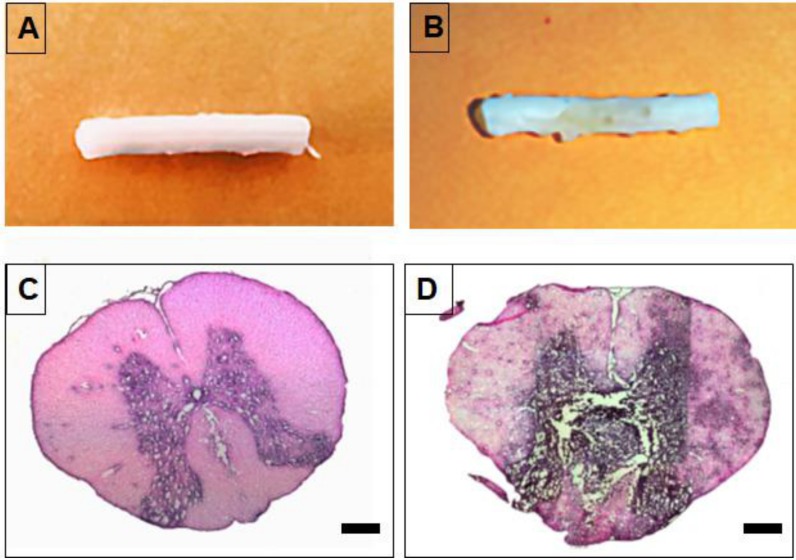
Generation of a rat T5 spinal cord tumor model at 12 days post-injection of C6 cells. (**A**,**B**) Representative images of isolated spinal cord and (**C**,**D**) H&E stained spinal cord section. (**A**,**C**) Normal spinal cord and (**B**,**D**) spinal cord with C6 cell-derived tumor. Scale bars indicate 500 µm.

**Figure 6:**
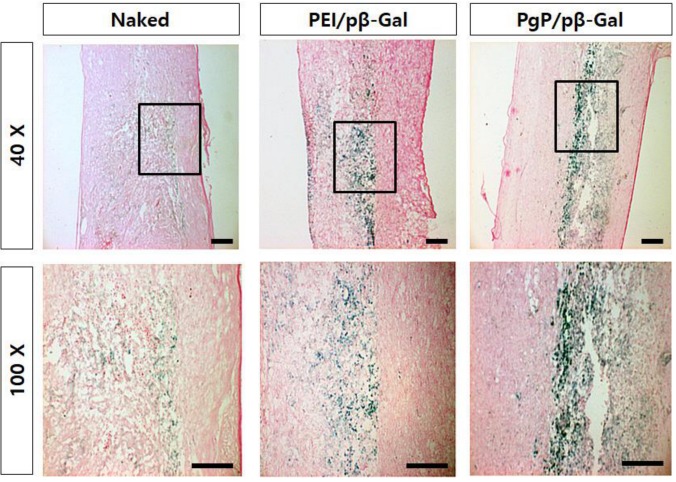
Representative images of β-Galactosidase-positive cells at 7 days post-injection of PgP/pβ-Gal polyplexes (N/P of 60/1) in rat T5 spinal cord tumor in vivo. (left) Naked pβ-Gal, (middle) bPEI/pβ-Gal polyplexes at N/P of 5/1, and (right) PgP/pβ-Gal polyplexes at N/P of 60/1. Original magnification: (**Top**) 40× and (**Bottom**) 100×. Scale bars indicate 100 µm.

To verify spinal cord tumor formation, rats were sacrificed at 12 days post-injection of C6 cells, tissues sectioned, and stained with H&E. The tumor masses were observed by high-density cell growth in between T5 and T6 in C6 cell-injected animals (Figure 5B,D), compared to normal spinal cord (Figure 5A,C). To evaluate the efficacy of PgP as a gene carrier in vivo, PgP/pβ-gal polyplexes (N/P 60/1, 10 μg) were intratumorally injected 5 days after C6 cell injection. Seven days later, the transfection efficiency of PgP/pβ-gal polyplexes in the spinal cord tumor was evaluated by x-gal staining. Figure 6 shows representative images of β-Gal-positive cells stained in blue within the spinal cord tumor area. We observed that the x-gal positively stained area in animals receiving PgP/pβ-gal polyplexes was substantially larger compared to animals receiving bPEI/pβ-gal polyplexes or naked pβ-gal (Figure 6).

### 3.7. Suicide Effect of PgP/pHSV-TK Polyplexes with GCV in a Rat Spinal Cord Tumor In Vivo

**Figure 7:**
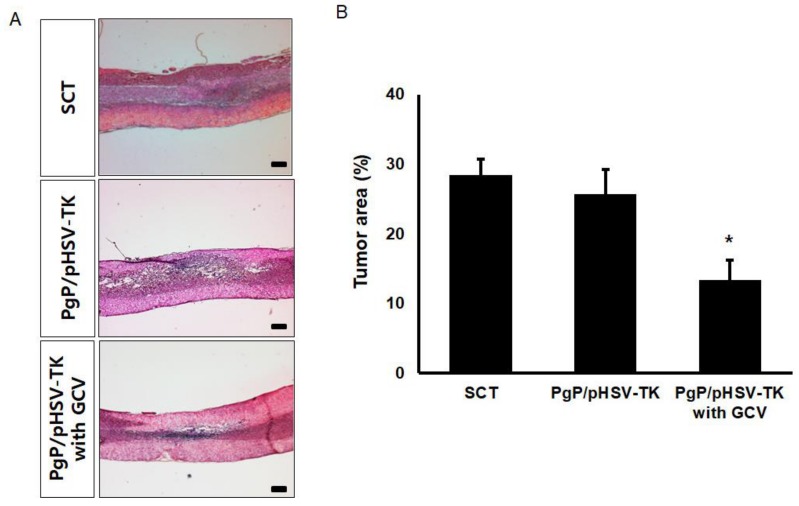
Histological analysis of the suicide effect on tumor size at 10 days post-intratumoral injection of PgP/pHSV-TK (10 µg pHSV-TK, N/P ratio of 60/1) polyplexes with GCV (40 mg/kg, intraperitoneal injection). (**A**) Representative images of H&E stained longitudinal spinal cord sections. Scale bars indicate 500 μm. (**B**) The percent tumor area of spinal cord tumors. The % tumor area was measured and averaged from 16 different sections of spinal cords from each group (4 sections/rat, 4 rats/ group). * *p* < 0.05 compared with untreated SCT group.

**Figure 8:**
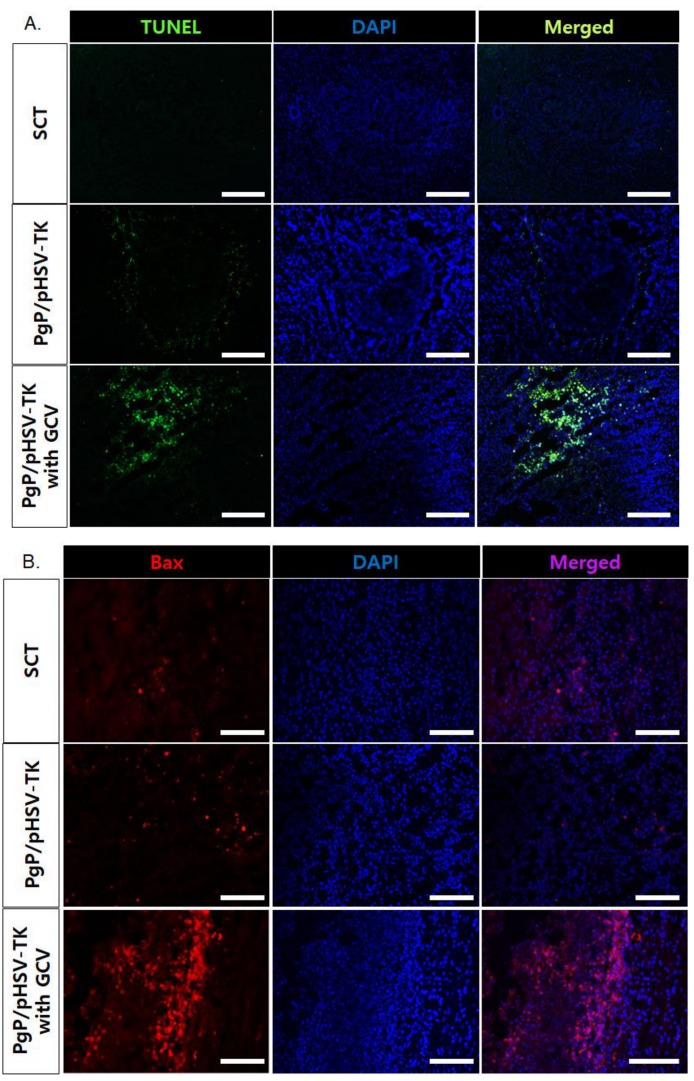
Representative images of (**A**) TUNEL+ cells (green), (**B**) Bax+ cells (red), in spinal cord tumor at 10 days post-intratumoral injection of PgP/pHSV-TK (10 µg pHSV-TK, N/P ratio of 60/1) polyplexes with GCV (40 mg/kg, intraperitoneal injection). Cell nuclei were counter stained with DAPI (blue). Scale bars indicate 100 μm.

To evaluate the suicide effect of PgP/pHSV-TK with GCV treatment, PgP/pHSV-TK polyplexes at an N/P ratio 60/1 (10 µg pHSV-TK) were injected into spinal cord tumors at 5 days post-injection of C6 cells and then received i.p. injection of 40 mg GCV/kg for 10 days. Figure 7A shows representative images of H&E stained spinal cord tumor sections from various animal groups. The percent tumor area in animals treated with PgP/pHSV-TK polyplexes and GCV injection was significantly lower than that of groups receiving PgP/pHSV-TK polyplexes alone and the untreated SCT group (Figure 7B). We also evaluated the effect of PgP/pHSV-TK with GCV treatment on apoptosis using the TUNEL assay and immunohistochemistry (IHC) for expression of the pro-apoptotic protein, Bax. The number of TUNEL-positive cells in spinal cord tumors was substantially higher in animals receiving PgP/pHSV-TK polyplexes with GCV injection than both the PgP/pHSV-TK polyplexes only group and the untreated spinal cord tumor group (Figure 8A). We also observed that Bax expression was highly upregulated in animals injected with PgP/pHSV-TK polyplexes plus GCV compared to both PgP/pHSV-TK polyplexes only and untreated spinal cord tumor groups (Figure 8B).

## 4. Discussion

Intramedullary spinal cord tumor (IMSCT) is an uncommon neoplasm that causes significant neurologic morbidity and mortality [2]. Surgical therapy, radiotherapy, and chemotherapy are currently the most common treatments [3,4,5]. In addition to these current treatments, gene therapy has been explored as a possible alternative approach to overcome some of the limitations associated with current therapies for SCTs [12,13].

A cationic, amphiphilic copolymer, PgP, has been developed by our group and has been shown to perform as an efficient drug and non-viral gene carrier in vitro as well as in *in vivo* animal models [21,22,24]. As a block copolymer that forms polymeric micelles in aqueous solution with a hydrophobic core and hydrophilic, cationic shell, PgP has the potential to serve as a combinatorial carrier for the simultaneous delivery of hydrophobic drugs and anionic therapeutic nucleic acids. Our long-term goal is to use PgP as a carrier for GCV and pHSV-TK co-delivery. In this study, we evaluated PgP as a gene carrier in glioma (C6) cells in vitro using reporter genes such as pGFP and a rat spinal cord tumor model in vivo using reporter gen, pβ-Gal, and therapeutic gene, pHSV-TK. We first evaluated the particle size and surface charge of PgP/pGFP polyplexes at various N/P ratios and found that PgP can form stable complexes with pDNA 138–148.5 nm in size and positive surface charge at all N/P ratios (Table 1). This size range and surface charge are suitable for intracellular uptake by endocytosis and avoidance of rapid clearance by the reticuloendothelial system (RES) in systemic delivery [25,26]. 

Barriers for systemic gene therapy in vivo are polyplex stability and degradation by nucleases in the blood stream. To model the stability of PgP/pGFP in the blood stream, we conducted a heparin competition study using negatively charged polysaccharide, heparin. PgP/pGFP at an N/P ratio of 60/1 showed high stability and heparin was only able to dissociate pGFP from PgP at 10 *w/w* ratio of heparin/pGFP and higher (Figure 1B). We also observed that PgP can efficiently protect pDNA from nucleases in serum for up to 24 h, whereas pDNA without PgP was completely degraded by serum nucleases within 3 h. These results show that PgP may be a promising non-viral gene carrier for systemic gene therapy *in vivo*. In the long-term storage stability study, we observed that the transfection efficiency of PgP/pGFP polyplexes in C6 (rat glioma) cells was maintained up to four months at 4 °C and this result is consistent with the long term stability of PgP/pGFP and PgP/siRhoA observed in B35 (neuroblastoma) cells in our laboratory [21,24]. This demonstrates that PgP can form a stable complex with both pDNA and siRNA and preserve the bioactivity of nucleic acids during long-term storage, an important challenge for the clinical translation of non-viral vectors.

To evaluate PgP as a pHSV-TK gene carrier, we administered two doses of GCV (50 and 100 µg/mL) after PgP/pHSV-TK transfection in C6 cells in vitro. The PgP/pHSV-TK transfected group with both GCV doses showed significantly higher anti-cancer efficacy compared to control groups including GCV only, PgP/pGFP only, and PgP/pHSV-TK without GCV treatment at both one and four days. The lack of a significant effect of GCV dose on the anti-cancer efficacy of PgP/pHSV-TK is consistent with results obtained for rPOA/HSV-TK transfection and GCV treatment by Won et al. [13]. We observed that the PgP/pHSV-TK transfection with GCV (100 µg/mL) treatment group showed significantly higher cell cytotoxicity compared to bPEI/pHSV-TK transfection with GCV (100 µg/mL) treatment group one day post-GCV treatment. In vivo β-Gal expression was substantially higher in animals receiving PgP/pβ-Gal polyplexes compared with bPEI/pβ-Gal polyplexes in the rat spinal cord tumor model. bPEI is known as an effective transfection reagent, being widely studied both in vitro and in vivo due to its high transfection efficacy and the ability of the proton sponge effect to facilitate endosomal escape [27,28]. However, poor transfection efficiency in the presence of serum and cytotoxicity are limitations to clinical application [29].

Finally, we evaluated the suicide effect of PgP/pHSV-TK with GCV in the rat spinal cord tumor model and observed that the percent tumor area in animals treated with PgP/pHSV-TK and GCV was significantly smaller than that of those receiving PgP/pHSV-TK polyplexes without GCV (Figure 7). This result was further confirmed by the presence of more TUNEL+ cells as well as increased expression of the pro-apoptotic protein, Bax, in the PgP/pHSV-TK polyplexes with GCV treatment group compared with the PgP/pHSV-TK without GCV group. These data demonstrate that PgP can serve as an effective pHSV-TK carrier to activate the prodrug GCV for the treatment of spinal cord tumors.

## 5. Conclusions

In this study, we demonstrated that the cationic amphiphilic copolymer PgP can be a gene carrier due to the stability of polyplexes in the presence of negatively charged serum proteins and the ability to protect pDNA from nucleases in the serum, PgP/pDNA polyplexes maintain bioactivity for transfection after storage at 4 °C for up to 4 months—an important feature for commercial and clinical application. We demonstrated that PgP can efficiently deliver pHSV-TK in C6 cells and PgP/pHSV-TK in combination with GCV showed significantly higher suicide effect on C6 cells compared to various control groups in vitro. Finally, we demonstrated that PgP/pHSV-TK with GCV treatment increases the suicide effect and apoptosis of tumor cells and reduces tumor size in a rat T5 spinal cord tumor model compared with PgP/pHSV-TK without GCV treatment. In the future, we will evaluate the potential of PgP as a GCV and pHSV-TK co-delivery carrier to improve the bioavailability and half-life of GCV as well as increase the survival rate after treatment of PgP/pHSV-TK with PgP-GCV in rat T5 spinal cord tumor model.

## Figures and Tables

**Table 1:** Mean particle size (PS), zeta potential (ZP), and polydispersity index (PDI) of PgP/pDNA polyplexes at various N/P ratios.

N/P ratio	15	30	45	60
Particle Size (nm)	141.2 ± 3.8	148.5 ± 3.8	138.0 ± 3.2	145.7 ± 1.5
PDI	0.17 ± 0.01	0.16 ± 0.01	0.20 ± 0.01	0.17 ± 0.01
Zeta potential (mV)	34.4 ± 0.2	41.3 ± 2.5	41.5 ± 0.7	41.5 ± 0.3
